# A novel approach to earlobe reconstruction using the V to Y advancement flap

**DOI:** 10.1186/s40463-021-00513-1

**Published:** 2021-05-19

**Authors:** Laura Allen, Kelti Munroe, S. Mark Taylor

**Affiliations:** 1 Division of Otolaryngology – Head & Neck Surgery, Department of Surgery, University of British Columbia, 2775 Laurel St, Vancouver, British Columbia V5Z 1M9 Canada; 2 Faculty of Medicine, Dalhousie University, 1459 Oxford Street, Halifax, Nova Scotia B3H 4R2 Canada; 3 Division of Otolaryngology – Head & Neck Surgery, Department of Surgery, QEII Health Sciences Centre, Dalhousie University, 3rd floor Dickson Building, 5820 University Avenue, Halifax, Nova Scotia B3H 1Y9 Canada

**Keywords:** Flap, Surgical, Ear auricles, Ear, Skin neoplasms, Aesthetics, Reconstructive surgical procedures

## Abstract

**Background:**

The V to Y advancement flap offers an excellent option for reconstructing defects of the lobule and adjacent structures of the external ear. We demonstrate its utility for small defects of the earlobe including those extending to the antitragal and conchal bowl regions. To our knowledge use of this technique for earlobe reconstruction has not been reported.

**Methods:**

A review of the literature was performed on the use of the V to Y flap for earlobe reconstruction. We then described its use in reconstructing lobular defects in 6 patients. All patients had a non-melanoma skin cancer involving the earlobe. All surgeries were performed under local anesthetic at a tertiary care centre in Halifax, Canada. Defects ranged in size from 1.0 to 1.4 cm. All defects were reconstructed with only a V to Y advancement flap. Patient photographs were taken intra-operatively and post-operatively. For all patients, satisfaction of the final aesthetic result was assessed on a 10 point scale in follow-up at 6 months.

**Results:**

A review of the literature did not reveal any reports of the V to Y flap used in isolation for lobular reconstruction. At our centre from 2018 to 2020, this method was well tolerated under local anesthetic in 6 patients with non-melanoma skin cancers of the earlobe. All patients reported an aesthetically satisfying result at 6 months with scores ranging between 8 and 10. Scarring in all cases was minimal.

**Conclusion:**

The V to Y advancement flap is a simple technique for reconstructing small defects of the lobule. This method is technically straight-forward, poses minimal risk to the patient, and in our experience, yields a favourable cosmetic outcome.

**Graphical abstract:**

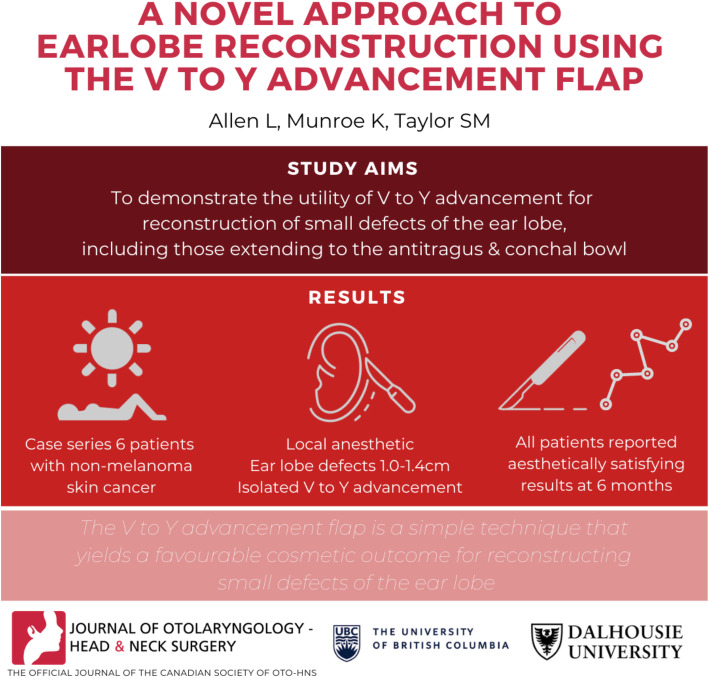

## Background

The V to Y advancement flap is a useful modality for repairing a variety of skin defects. Its use is well described in the literature. The flap is designed to transfer skin from an area of relative excess to fill a neighbouring defect. It is a versatile local advancement flap that is technically straightforward and can vary markedly in size. Surgical technique involves making a “V” shaped incision down to subcutaneous fat, adjacent to the defect. The “V” shaped tissue is then advanced to cover the defect. The resulting donor site is sutured in a straight line which creates a “Y” shaped closure. Blood supply of the external ear is from the posterior auricular and superficial temporal branches of the external carotid artery. The superior auricular artery bridges these two arteries to form a dependable collateral blood supply. These vessels consistently provide several small perforating vessels that keep the lobule, and consequently the V to Y flap, well vascularized [1]. Additionally, the anterior auricular branch of the superficial temporal artery as well as the occipital artery contribute to arterial supply to the external ear. The V to Y flap has many indications, across many surgical disciplines. Examples include its use in reconstructing facial defects; often after skin cancer excision and in cosmetically sensitive areas of the mouth and nose. It is also described in upper and lower limb reconstruction, and it is commonly used in the repair of sacral ulcers, and in gluteal and vulvar reconstructions. The flap is perhaps most notably described in fingertip reconstruction. However, its use in ear reconstruction is not well documented in the literature. More commonly, reconstruction of the earlobe is described in other contexts such as repair of congenital malformations, including Pixie lobe deformities, or in keloid scar revisions. While cutaneous malignancies commonly present on the external ear, it is often larger lesions, which involve more complex reconstructions, that have been reported.

The head and neck are areas of high sun exposure, and consequently, are among the most common locations for the development of non-melanoma skin cancers, particularly in those with fair skin, light eyes, or Celtic ancestry [2]. The “H-zone” of the face is an area where cutaneous skin cancers are likely to occur. This area is a mask-shaped distribution that corresponds with overlying embryonic fusion plates. It includes the upper lip, the junction of the ala with the nasolabial fold, the nasal ala, the septum, the inner canthi and lower eyelids, the periauricular region extending to the temple, and certain scalp regions. Auricular basal and squamous cell carcinoma that are greater than 0.6 cm on the facial H-zone are considered high risk lesions for recurrence and metastasis [3]. Moreover, the external ear is an aesthetically challenging area given its complex cartilaginous contours and adherent, thin overlying skin. This makes the lobule an advantageous source of loose tissue that can be mobilised to reconstruct external ear structures. To our knowledge, use of the V to Y flap for lobular reconstruction of small skin cancers has not been described in the literature. In the context of non-melanoma skin cancers, which are the most common cancer diagnosis in Canada [4], we believe that awareness of this reconstructive technique is beneficial to surgeons who will likely encounter facial skin cancers in practice. This technique may impact the practice of a wide international target audience of clinicians who perform excision and reconstruction of auricular defects in practice, including but not limited to dermatologists, plastic and facial plastic surgeons, otolaryngologist-head and neck surgeons, as well as general practitioners, particularly those who may perform outpatient procedures in more rural settings. We aim to describe the use of the V to Y flap in 6 Caucasian patients with non-melanoma skin cancer of the earlobe.

## Methods

First, a review of the literature was performed using Google Scholar and PubMed. Terms searched included “ear”, “reconstruction”, “defect”, “lobule”, “non-melanoma”, “skin cancer”, “VY”, and “advancement flap”. No date range was defined. Inclusion criteria were studies published in English. Exclusion criteria were those studies that did not describe surgery on the ear, did not mention a “VY” reconstruction, or were not published in English. The initial search yielded 90 results. Duplicates were removed and titles were screened against exclusion criteria. This yielded 20 remaining results. Articles were scrutinized further and those that described reconstruction of the external ear using cartilage grafts, larger transposition flaps, or wedge resections were excluded. None of the resulting articles described the use of the V to Y flap in isolation for reconstructing lobule defects. Articles were also hand searched by two authors with an emphasis on “VY” and “ear reconstruction”. Again, this did not reveal any articles that specifically described the use of the V to Y flap for lobule reconstruction.

At a tertiary care centre in Halifax, Canada, 6 Caucasian male patients ranging in age from 72 to 85 underwent primary resection of non-melanoma skin cancer of the earlobe ranging in size from 1.0–1.4 cm in maximum diameter. All lesions involved the lobule, with minor involvement in some cases of the conchal bowl and antitragal regions. All remaining defects underwent subsequent reconstruction using simply the V to Y subcutaneous island advancement flap. Surgeries were performed by the senior author. Photographs were taken intraoperatively and postoperatively. All surgeries were performed under local anaesthesia in an outpatient setting. Closures were performed deeply with monocryl and skin was closed using a 5.0 plain gut suture in an interrupted fashion. Aesthetic results were analyzed in follow-up at 1 month and again at 6 months. Patients were asked to rate their aesthetic satisfaction at 6 months postoperatively on a 10-point scale, with 10 representing “best possible outcome” and 0 representing “unsatisfied with result”. The Likert-type 10-point scale was chosen for the following reasons. Firstly, *aesthetic satisfaction* was the sole outcome of interest. Patient-Reported-Outcome-Measures (PROM) scales use multiple outcome measures, often focusing on health-related quality of life measures which were not the aim of our study. Secondly, we felt that validated ear-specific surveys such as the EAR-Q scale, while appropriate for surveying Otoplasty patients, have a broad set of measures that are beyond aesthetic evaluation of the lobule alone [5]. Furthermore, the Likert-type format is one of the most widely used scales in the social sciences. Its validity and reliability have been widely debated, yet it has been found that as the size of a Likert scale increases, validity and reliability may also increase [6]. Given the subjective nature of our outcome of interest, as well as our limited outcome measurements, we chose to employ the 10-point Likert scale.

## Results

**Fig. 1:**
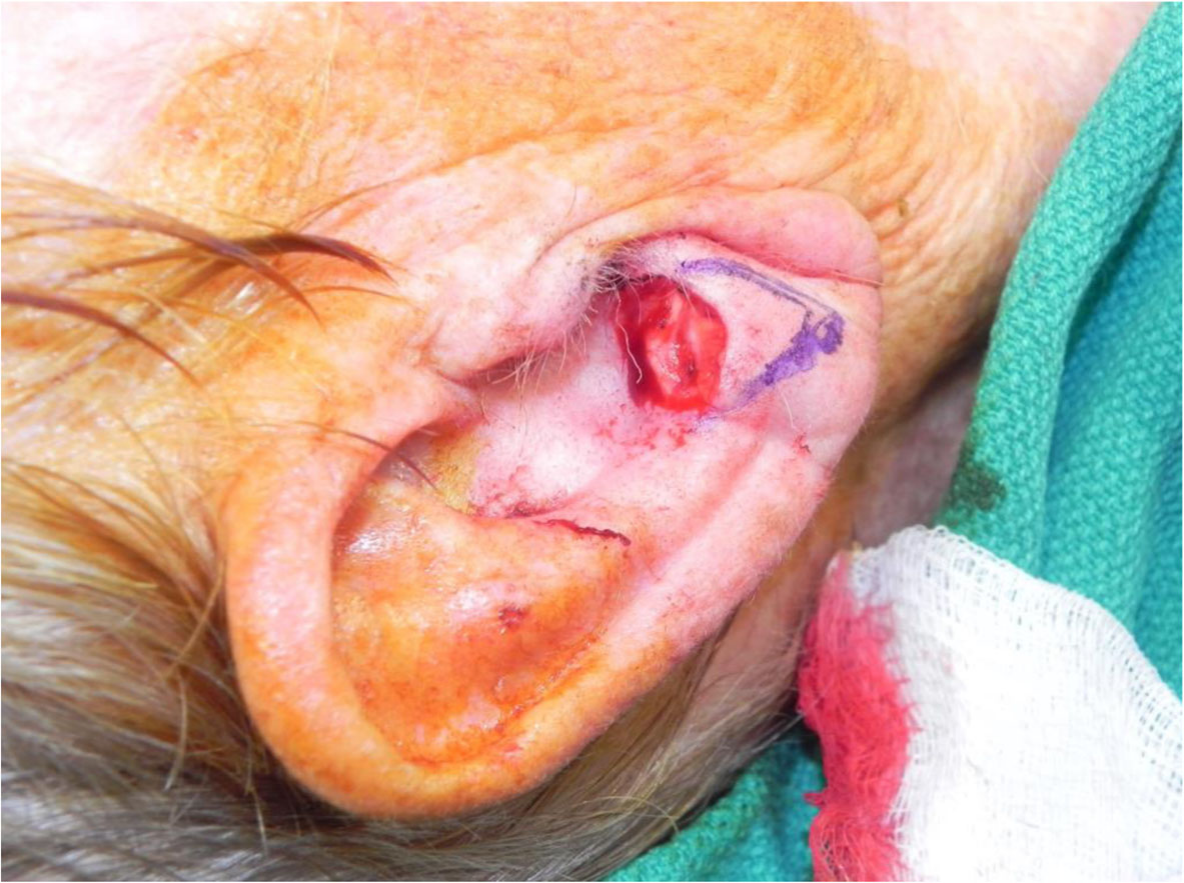
Patient no. 1. intraoperative defect following excision

**Fig. 2:**
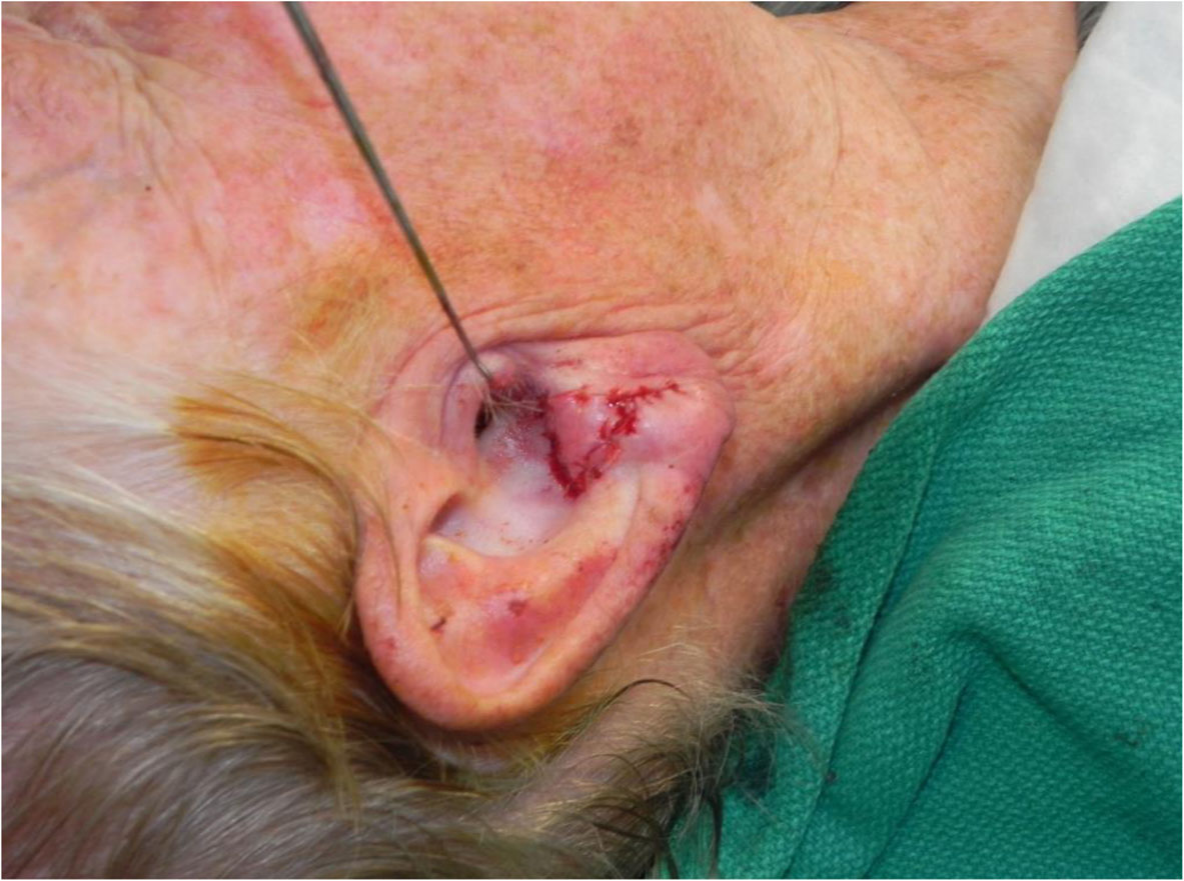
Patient no. 1. intraoperative following V to Y reconstruction

**Fig. 3:**
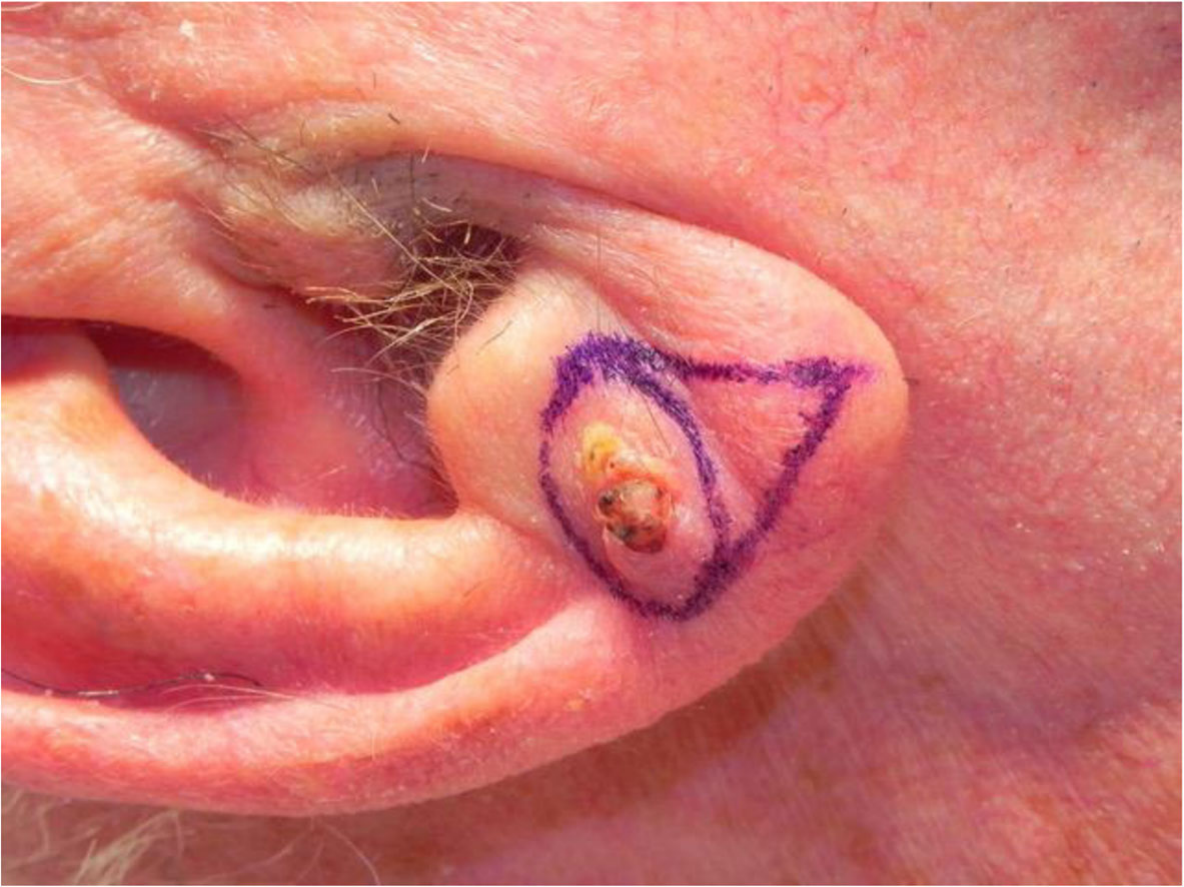
Patient no. 2. intraoperatively prior to lesion excision

**Fig. 4:**
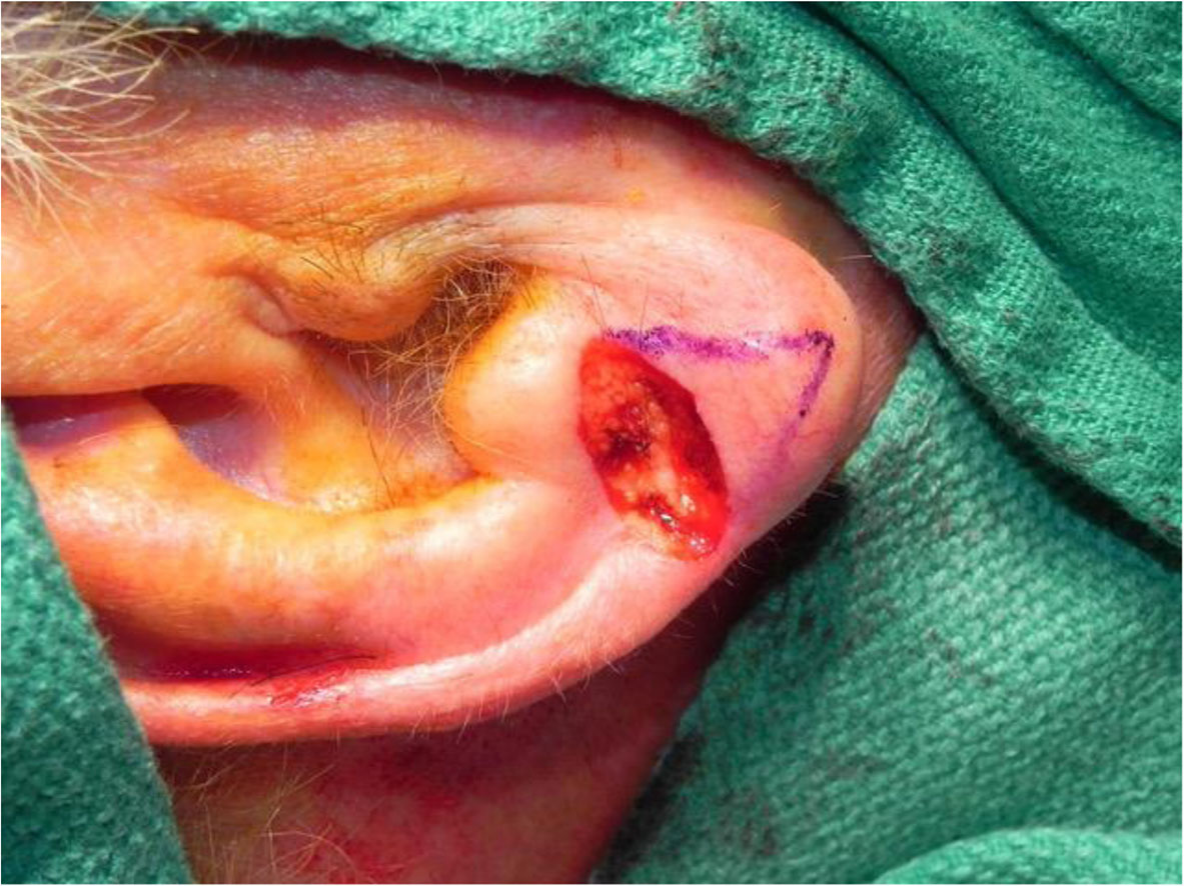
Patient no. 2. intraoperative defect following excision

**Fig. 5:**
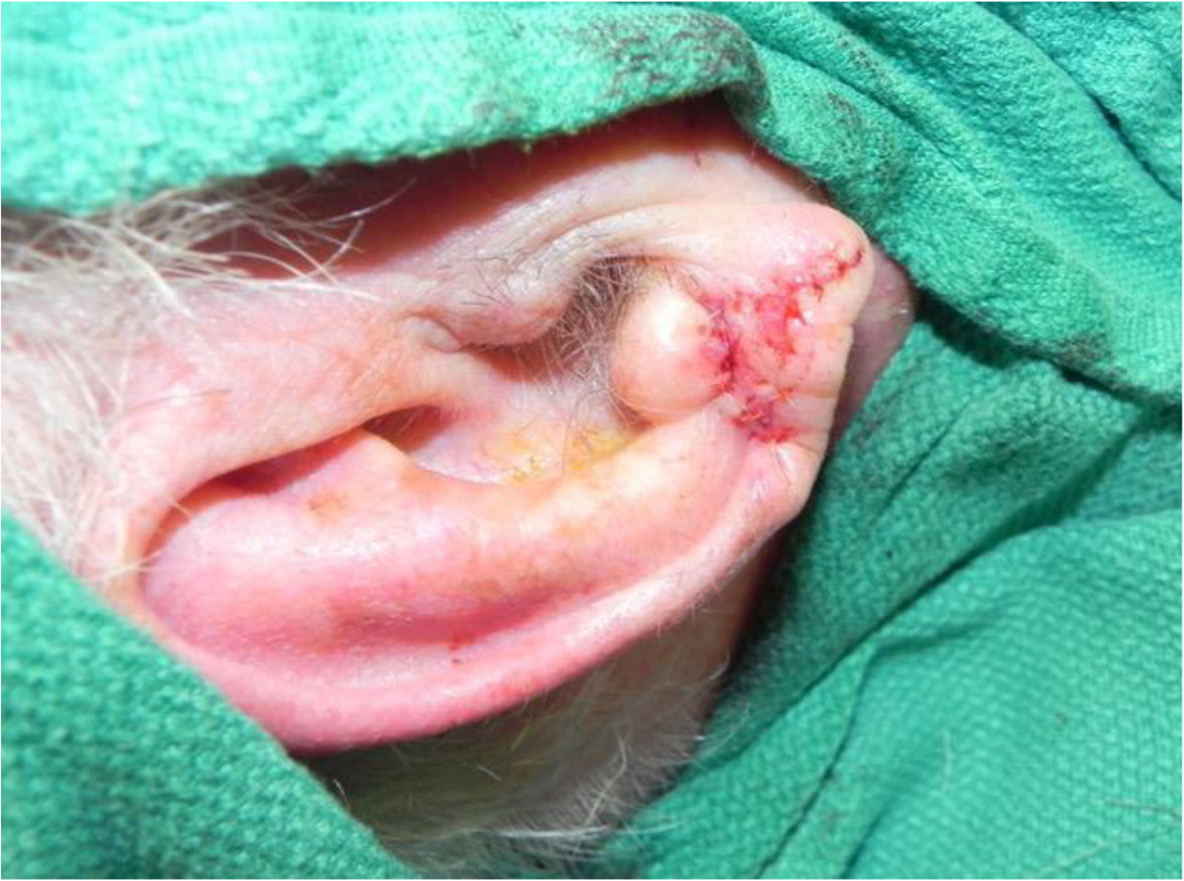
Patient no. 2. intraoperative following V to Y reconstruction

**Fig. 6:**
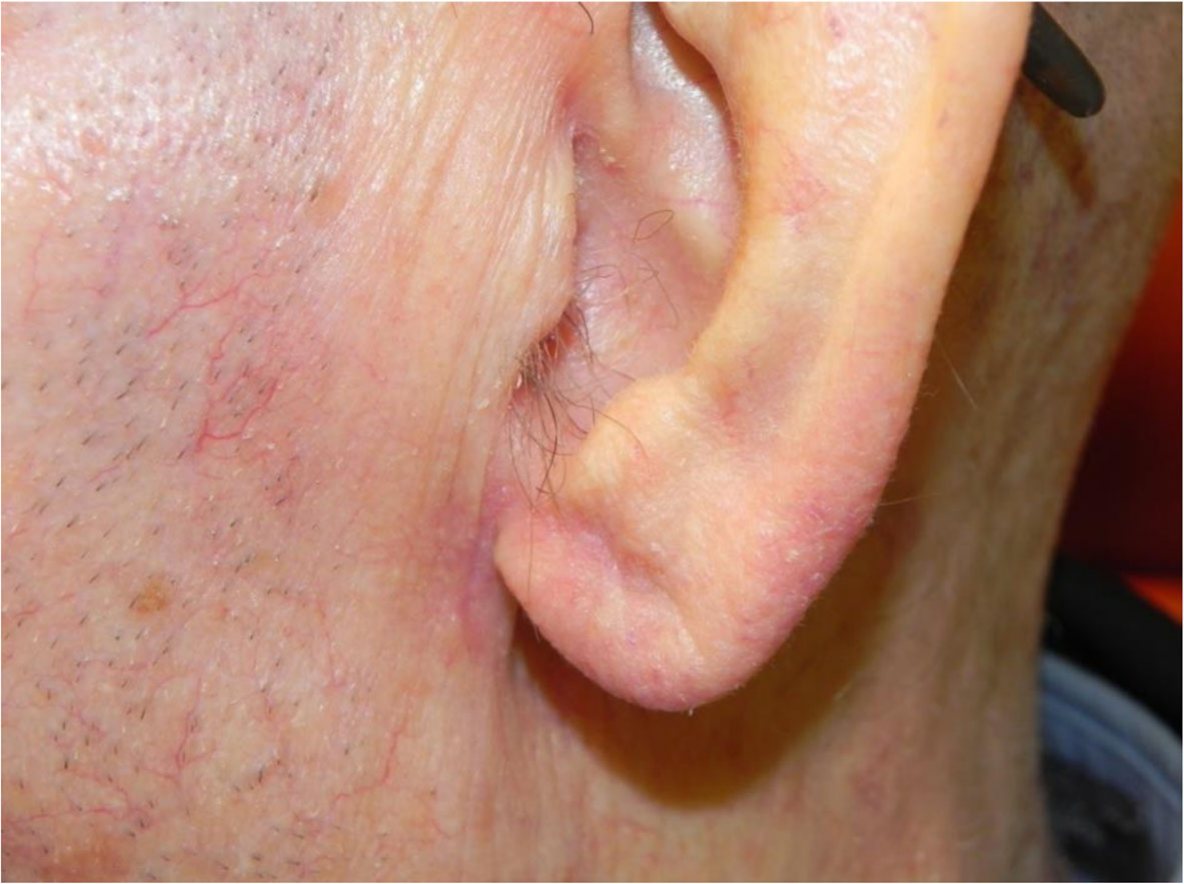
Patient no. 2. postoperative result at 6 month follow-up

**Fig. 7:**
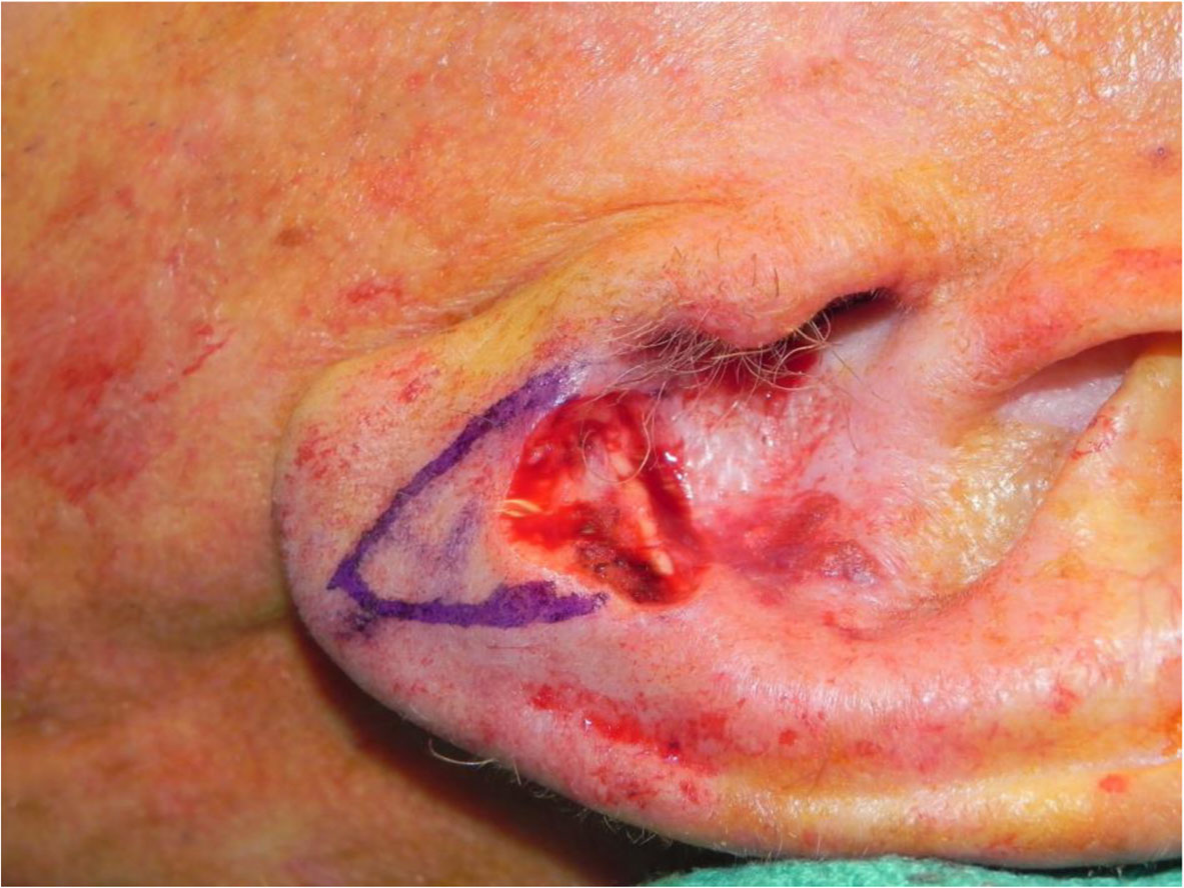
Patient no. 3. intraoperative defect following excision

**Fig. 8:**
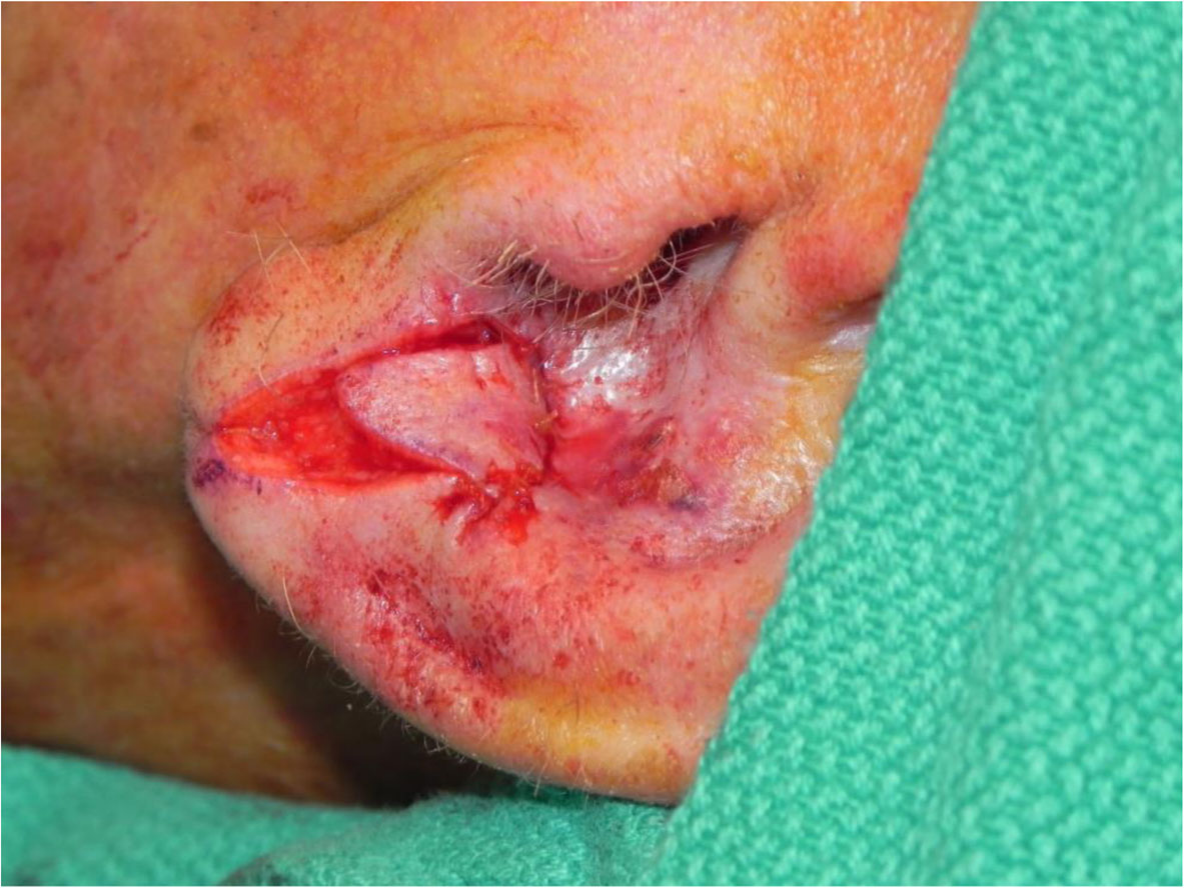
Patient no. 3. intraoperative defect during V to Y reconstruction

A review of the literature did not reveal any studies that reported the isolated use of the V to Y flap for lobular reconstruction. At our centre from 2018 to 2020, 6 patients underwent excision of a 1.0–1.4 cm lesion of the lobule with advancement of a V to Y flap in the superior direction over the defect (Figs. 1, 2, 3, 4, 5, 6, 7 and 8). This was well tolerated under local anesthetic in all cases. No intraoperative or postoperative complications occurred including infection or bleeding. Final pathology confirmed that 5 lesions were BCC and 1 was SCC: all with negative margins. There were no cases of hypertrophic scarring. Scars in all cases were fine line and minimal. All patients reported an aesthetically satisfying result at 6 months with scores ranging between 8 and 10. Specifically, 3 patients reported a score of 8, 2 a score of 9, and 1 was reported as 10 (Table 1).

**Table 1:** Patient aesthetic satisfaction scores on a 10 point scale

	10/10	9/10	8/10
**Patient 1**			**1**
**Patient 2**		**1**	
**Patient 3**	**1**		
**Patient 4**			**1**
**Patient 5**			**1**
**Patient 6**		**1**	
**Total**	**1**	**2**	**3**

## Discussion

In our experience, the V to Y advancement flap when used for the reconstruction of lobular defects is a relatively simple surgical technique that is well-tolerated under local anesthetic in the outpatient setting. Of note, patient selection is important for employment of this approach. Patients with a tendency for keloid or hypertrophic scarring, congenital external ear malformations such as microtia with limited lobule tissue, or ears that have been previously operated on may not be ideal candidates. This technique is indicated for smaller lesions that involve the lobule and surrounding regions. Its use is limited for larger defects that involve the majority of the lobule and leave little tissue for advancement, or for lesions that are more extensive and involve surrounding cartilage and periauricular structures. Similarly, it is not indicated for aggressive carcinomas or in patients with recurrences that warrant greater tissue margins. However, when indicated, this technique is advantageous from an aesthetic perspective, resulting in fine line scars that compliment the natural creases of the lobule. Our study is limited to the participation of 6 patients, all of whom are Caucasian. This small sample size may influence validity of results. Additionally, a larger more diverse population sample could strengthen findings, and potentially influence scarring results. Additionally, all specimens in our study had clear margins on final pathology. However, in the case of a positive margin which requires further resection, this technique could allow for repeated resection without having initially lost a significant amount of transferable tissue which may have been sacrificed using another technique, such as a wedge resection. Another advantage of this technique is that it minimizes procedural length and recovery for patients in comparison to approaches that involve more complex reconstructions and larger tissue transfers and complicated post operative ear dressings. The donor defect is aesthetically negligible and results in no vertical shortening of the lobule itself, maintaining symmetry with the unoperated ear. Importantly, in our experience, patients reported high satisfaction rates with their cosmetic results over the 6 month postoperative period. Overall, this is a useful technique that can benefit both surgeon and patient. It is time and cost effective and benefits the patient while avoiding the risk of general anesthetic.

## Conclusion

Use of the V to Y advancement flap for reconstructing defects of the earlobe is a novel yet simple technique that is technically straight-forward, poses minimal risk to the patient, can be performed in an outpatient setting, and in our experience, yields a favourable cosmetic outcome.

## Data Availability

Included in article.
